# Pentoxifylline adjunct to risperidone for negative symptoms of stable schizophrenia: a randomized, double-blind, placebo-controlled trial

**DOI:** 10.1093/ijnp/pyae051

**Published:** 2024-12-24

**Authors:** Ahmad Shamabadi, Elham-Sadat Rafiei-Tabatabaei, Kimia Kazemzadeh, Kimia Farahmand, Bita Fallahpour, Mohammad-Reza Khodaei Ardakani, Shahin Akhondzadeh

**Affiliations:** Psychiatric Research Center, Roozbeh Psychiatric Hospital, Tehran University of Medical Sciences, Tehran, Iran; Psychiatric Research Center, Roozbeh Psychiatric Hospital, Tehran University of Medical Sciences, Tehran, Iran; Psychiatric Research Center, Roozbeh Psychiatric Hospital, Tehran University of Medical Sciences, Tehran, Iran; Psychiatric Research Center, Roozbeh Psychiatric Hospital, Tehran University of Medical Sciences, Tehran, Iran; Department of Psychiatry, Razi Hospital, University of Social Welfare and Rehabilitation Sciences, Tehran, Iran; Department of Psychiatry, Razi Hospital, University of Social Welfare and Rehabilitation Sciences, Tehran, Iran; Psychiatric Research Center, Roozbeh Psychiatric Hospital, Tehran University of Medical Sciences, Tehran, Iran

**Keywords:** combination drug therapy, neuroimmunomodulation, psychosis, psychotic disorder, randomized controlled trial

## Abstract

**Background:**

Negative symptoms of schizophrenia represent an unmet therapeutic need for many patients in whom pentoxifylline may be effective in terms of its dopaminergic, anti-inflammatory, and cerebral blood flow–increasing properties. This study aimed to evaluate pentoxifylline as a therapeutic agent for improving negative symptoms of schizophrenia.

**Methods:**

Chronic schizophrenia outpatients experiencing significant negative symptoms were randomly allocated to receive pentoxifylline 400 mg or matched placebo every 12 hours for 8 weeks. All patients were clinically stable as they had received risperidone for at least 2 months, which was continued. The participants were assessed using the Positive and Negative Syndrome Scale (PANSS), Hamilton Depression Rating Scale, Extrapyramidal Symptom Rating Scale, and side effect checklist.

**Results:**

The patients’ baseline characteristics were comparable between the groups. There was a significant time–treatment interaction effect on PANSS negative subscale scores ($\eta_{P}^{2}$=0.075), with the pentoxifylline group showing significantly greater reductions until weeks 4 (Cohen *d* = 0.512) and 8 (Cohen *d* = 0.622). Also, this group showed a significantly better response by week 8. Other PANSS scores, Hamilton Depression Rating Scale scores, Extrapyramidal Symptom Rating Scale scores, and side effect frequencies were comparable between the groups. Pentoxifylline showed a nonsignificant higher remission of 37.1% compared with 14.7% in the placebo group.

**Conclusions:**

Pentoxifylline was safely and tolerably beneficial for the primary negative symptoms of chronic schizophrenia.

**Significance statement**

With an appropriate design to assess primary negative symptoms—by initially stabilizing patients and controlling for secondary factors—and precise baseline characteristic adjustments, this study introduces pentoxifylline as a potentially tolerable adjunctive treatment in treating primary negative symptoms in patients with chronic schizophrenia. Current pharmacotherapy strategies have issues with efficacy and complications. Achieving these effects in this short period for a disorder with significant burden and treatment limitations is of great interest.

## INTRODUCTION

Current treatments for schizophrenia, including antipsychotic medications, long-acting injectable antipsychotics, and psychotherapy, are efficient for almost two-thirds of patients. Patients often require long-term treatment with significant side effects (Akhondzadeh, 2001; Patel et al., 2014; Leung et al., 2019), and a substantial proportion of patients are resistant to treatment (Farmer and Blewett, 1993). Moreover, treatments primarily target positive symptoms and are less effective in treating negative symptoms (Stępnicki et al., 2018). The burden of negative symptoms on individuals and society is substantial, making effective treatment crucial for improving overall well-being (Sarkar et al., 2015; Cerveri et al., 2019). Researchers are trying to develop approaches to overcome this resistance, one of which is adjunctive therapy, considering the neuropathophysiology (Abbasi et al., 2010).

Dopamine dysregulation has been implicated in the pathophysiology of negative symptoms. Reduced striatal dopamine synthesis capacity has been found to be associated with negative symptoms. Also, studies have shown that reduced ventral striatal activity during reward anticipation is a cross-diagnostic finding in depression and schizophrenia, highlighting the importance of reward processing in understanding negative symptoms (Noorbala et al., 1999; Bègue et al., 2020; Galderisi and Kaiser, 2023). The failure to sufficiently improve these symptoms after treatment may be attributed to negative symptoms not being primarily driven by dopamine dysregulation, which is the primary target of antipsychotics (Sarkar et al., 2015). Increased levels of pro-inflammatory cytokines such as interleukin (IL)-1β and tumor necrosis factor (TNF)-α have been found in the cerebrospinal fluid of schizophrenia patients, particularly those with negative symptoms. Additionally, studies have shown that neuroinflammation can lead to the activation of microglia, which can contribute to the disruption of frontostriatal circuits and the development of negative symptoms (Bègue et al., 2020; Galderisi and Kaiser, 2023). Moreover, abnormalities in glutamate receptors and release patterns may contribute to the pathophysiology of negative symptoms (McCutcheon et al., 2020).

Pentoxifylline is a vasoactive medication that enhances blood circulation by lowering blood viscosity, which is indicated in the treatment of claudication (Bowton et al., 1989; Annamaraju et al., 2024). It is also used for venous ulcers, severe alcoholic hepatitis, and osteoradionecrosis (Annamaraju et al., 2024). Pentoxifylline is a methylxanthine derivative that inhibits the activity of phosphodiesterases (PDEs), particularly PDE3 and PDE4 (Neves et al., 2015). It has been shown that pentoxifylline can reverse depressive behavior in animal models, suggesting an antidepressant-like effect. This effect may be related to the increased levels of cyclic adenosine monophosphate (cAMP) and cyclic guanosine monophosphate within cells, which can modulate neurotransmitter release and synaptic plasticity (Bah et al., 2011). In addition, pentoxifylline reduces the expression of pro-inflammatory cytokines, for example, TNF-α, and attenuates the activation of inflammatory cells, thereby decreasing the neuroinflammatory response. The increased levels of cAMP and cyclic guanosine monophosphate may enhance the release of neurotransmitters such as dopamine. Also, its antioxidant properties may help protect neurons from oxidative damage, which is a key contributor to neurodegeneration in Parkinson disease (Neves et al., 2015; Bègue et al., 2020; Galderisi and Kaiser, 2023). Pentoxifylline may be effective as a therapeutic approach for negative symptoms in schizophrenia through the aforementioned mechanisms.

Research has shown that pentoxifylline significantly attenuates the increased time spent in hyperactive and restless behaviors induced by dexamethasone in rats, suggesting an anti–manic-like effect (Nassar and Azab, 2022). More specifically, a randomized, double-blind, placebo-controlled clinical trial showed that it can help reduce schizophrenia cognitive deficits and alleviate psychotic symptoms (Sinichi et al., 2023). This study aimed to evaluate pentoxifylline as a therapeutic agent for improving negative symptoms of schizophrenia.

## MATERIALS AND METHODS

The protocol of this trial was prospectively registered to the Iranian Registry of Clinical Trials (https://irct.behdasht.gov.ir/), a Primary Registry in the World Health Organization Registry Network, on May 26, 2022 (identifier: IRCT20090117001556N143). The Consolidated Standards of Reporting Trials framework was used in designing, conducting, and reporting (Appendix 1) (Schulz et al., 2010).

### Design and Setting

This clinical trial was conducted using an 8-week, parallel-group, randomized, double-blind, placebo-controlled design in chronic schizophrenia patients in the outpatient clinic of Roozbeh Hospital and Razi Hospital, 2 large-scale psychiatry hospitals affiliated with Tehran University of Medical Sciences and University of Social Welfare and Rehabilitation Sciences, respectively, from June 2022 to June 2024.

### Ethics

The ethical principles of the Declaration of Helsinki (Association, 2013) were observed in design and conduct. The School of Medicine Ethics Committee, Tehran University of Medical Sciences, approved the study protocol on March 19, 2022 (identifier: IR.TUMS.MEDICINE.REC.1401.159). The patients were informed that they could withdraw their consent to participate in the trial at any time, for any reason, without affecting their standard treatment or relationship with the service providers.

### Participants

Patients aged between 18 and 60 years who had been diagnosed with schizophrenia for at least 2 years and received risperidone for at least 2 months with no indication for hospitalization were eligible. The diagnosis was confirmed by a senior psychiatrist using the DSM-5 (First et al., 2015). No antipsychotic regimen was altered for a patient to be included. Eligible patients had a negative subscale score of >15 on the Positive and Negative Syndrome Scale (PANSS) (Kay et al., 1987) and a total score of <14 on the Hamilton Depression Rating Scale (HDRS) (Hamilton, 1960). Patients needed to be clinically stable, with a PANSS score change of ≤20% over 2 consecutive visits spaced 2 weeks apart while receiving a stable dose of risperidone (Salehi et al., 2022).

Patients with systemic diseases, lactation or pregnancy, suicidal ideation, alcohol or substance use disorder within the past 6 months, history of head trauma causing cerebral or retinal bleeding, history of neurosurgery, history of electroconvulsive therapy in the past 3 months, history of hypersensitivity to xanthine derivatives, or an intelligence quotient (Stern, 1949) of <70 were not included. Furthermore, the occurrence of severe side effects and the use of mood stabilizers, antidepressants, other antipsychotics, antihistamines, and supplements during the trial also disqualified participants.

### Intervention Arms

All participants maintained their ongoing risperidone (Risperdal; Janssen Pharmaceuticals, Beerse, Belgium), taking 4 to 6 mg/d orally. Additionally, they were allocated to receive oral capsules of pentoxifylline (EXIR Pharmaceutical Company, Tehran, Iran) 400 mg or matched placebo every 12 hours for 8 weeks.

Dosette boxes were supplied to participants every 4 weeks to enhance medication adherence. Compliance was monitored through patient and family member reports and counting returned capsules. If participants demonstrated noncompliance, defined as >30% of capsules being returned or reported instances of forgetting to take medication, they were not provided with additional medication but continued with all other aspects of the protocol.

As mentioned, using other interventions was strictly limited, except in cases of extrapyramidal symptoms, where biperiden was allowed orally up to 6 mg/d.

### Sample Size

Enrolling 37 participants in each arm was deemed sufficient to detect a between-group difference of 3.5 on PANSS negative subscale score reduction with an SD of 4 using a 2-tailed *t* test of mean differences assuming 90% power, 5% significance level, and 20% dropout rate and enhancing the generalizability of results.

### Randomization, Allocation Concealment, and Blinding

Independent individuals were responsible for randomization, allocation concealment, and blinding procedures. The participants were randomly allocated to the groups with a 1:1 ratio applying the permuted block technique with a block size of 4. Assignment numbers were stored in sequentially numbered, sealed, opaque envelopes to ensure concealment. Moreover, the identical appearance, texture, and sensory characteristics of pentoxifylline and placebo capsules maintained the blinding of participants, care providers, and outcome assessors.

### Tools and Assessments

Data on gender, age, marital status, literacy level, and disorder duration were collected at the initial assessment.

Schizophrenia psychopathological symptoms were assessed using the PANSS at baseline and weeks 4 and 8. The PANSS is a well-established and widely used tool for evaluating symptoms in schizophrenia, comprising subscales for positive (7 items), negative (7 items), and general psychopathology (16 items) symptoms. Each item is scored on a 1 to 7 Likert scale (Kay et al., 1987). Before allocation, a review of educational clinical slides on the PANSS and a discussion of applying assessment details were done during a meeting of the 2 outcome assessors with the principal investigator to familiarize them further with this tool, which resulted in an inter-rater reliability of >90%. Additionally, the HDRS, a widely accepted and standardized tool for assessing depression, was employed to assess depressive symptoms at baseline and week 8. These have been extensively used in clinical trials to assess outcomes in Iranian patients with schizophrenia (Salehi et al., 2022; Shamabadi et al., 2023).

Moreover, the Extrapyramidal Symptom Rating Scale (ESRS), a widely employed tool in clinical trials on Iranian patients (Salehi et al., 2022; Shamabadi et al., 2023), was employed to evaluate drug-induced parkinsonism, tardive dyskinesia, dystonia, and akathisia (Chouinard and Margolese, 2005) at baseline and week 8. Other side effects were assessed using open-ended questions and a checklist, enabling patients and their families to report any unlisted side effects (Akhondzadeh et al., 2003; Gougol et al., 2015; Emadi-Kouchak et al., 2016). A 24-hour hotline was available for patients to report any complications as they arose.

### Outcomes

The primary outcome was group differences in PANSS negative subscale score changes from baseline to endpoint. Comparing response rates at weeks 4 and 8 (defined as at least a 25% reduction in PANSS negative subscale scores; Leucht et al., 2007), the time needed to respond, and PANSS negative subscale score changes from baseline to week 4, as well as PANSS positive and general psychopathology subscale and total score changes from baseline to each time point, remission rate at week 8 (defined as a mean score of ≤2 in each subscale; Yen et al., 2002), HDRS score changes, ESRS score changes, and frequency of side effects between the groups were the secondary outcomes.

### Statistical Analyses

The Statistical Package of Social Science Software 27 (IBM, Armonk, NY, USA) was used for statistical analyses. A significance level of 0.05 was used. The Shapiro-Wilk test and Q-Q probability plots confirmed that continuous variable distributions did not significantly differ from normality. Analyses included the participants who completed the trial or withdrew after week 4 except in side effect frequency, which included all participants who received even 1 dose of pentoxifylline or placebo.

Qualitative variables, including gender, marital status, literacy level, response rate, and side effect frequencies, were reported as numbers (*n*) and percentages (%) and were compared using the chi-square test. The response time was compared between the groups using the Kaplan-Meier estimate with the log-rank test. The censoring rules were the participants’ withdrawal and termination of the study before the occurrence of the actual event to the participants. Quantitative variables were presented as mean ± SD and were compared using the 2-tailed, independent-samples *t*-test. Differences in PANSS, HDRS, and ESRS score changes from baseline to each timepoint between the groups were reported as mean difference (MD) with a 95% CI. Cohen *d* was used to measure effect sizes for the independent-samples *t*-test. The conventional thresholds are 0.2, 0.5, and 0.8, indicating small, moderate, and large sizes, respectively (Lakens, 2013). The general linear model repeated-measures ANOVA evaluated time and time–treatment interaction effects on PANSS scores. The results of multivariate tests were reported using Phillai’s Trace, and the results of tests of within-participants effects were corrected in nonspherical cases using Greenhouse-Geisser when ε was <0.75. Partial *eta* squared (ηP2) was implemented for effect sizes of 1-way repeated-measures ANOVA, with 0.0099, 0.0588, and 0.1379 indicating small, moderate, and large sizes, respectively (Lakens, 2013).

## RESULTS

**Figure 1.**
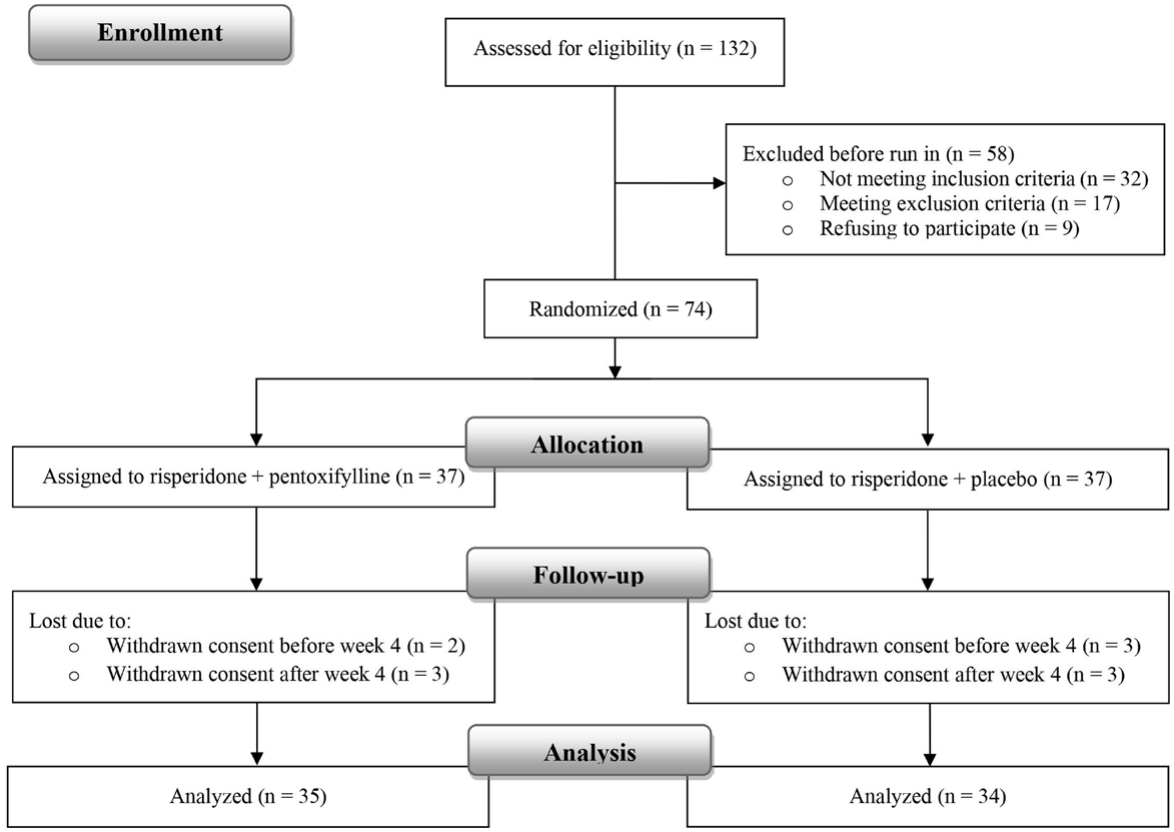
Flow chart presenting participant selection for the trial program and data analyses.

The flow diagram in **Figure 1** illustrates the selection process as well as the reasons for participant dropouts. The patients’ baseline demographic and clinical characteristics included in the efficacy analyses are reported and compared in **Table 1**.

**Table 1.** The baseline characteristics of the patients.

Variable		Risperidone + pentoxifylline (*n* = 35)	Risperidone + placebo (*n* = 34)	*P* ^#^
Gender, n (%)	Male	20 (57.1%)	18 (52.9%)	0.811^a^
	Female	15 (42.9%)	16 (47.1%)	
Age, mean y ± SD		36.63 ± 6.94	36.21 ± 7.09	0.803^b^
Marital status, n (%)	Single	11 (31.4%)	16 (47.1%)	0.311^c^
	Married	21 (60.0%)	17 (50.0%)	
	Separated	3 (8.6%)	0 (2.9%)	
	Widow	0	0	
Literacy level, n (%)	Illiterate	1 (2.9%)	3 (8.8%)	0.685^c^
	Primary school	3 (8.6%)	2 (5.9%)	
	Secondary school	10 (28.6%)	11 (32.4%)	
	Diploma	11 (31.4%)	12 (35.3%)	
	Higher education	10 (28.6%)	6 (17.6%)	
Disorder duration, mean years ± SD		14.60 ± 7.44	12.38 ± 6.76	0.200^b^
PANSS, mean scores ± SD	Negative	23.57 ± 7.24	24.06 ± 7.90	0.790^b^
	Positive	20.83 ± 8.20	20.68 ± 8.30	0.939^b^
	General psychopathology	40.66 ± 5.23	39.53 ± 6.30	0.421^b^
	Total	85.03 ± 10.24	84.35 ± 9.79	0.780^b^
HDRS, mean scores ± SD		8.14 ± 1.57	8.03 ± 1.31	0.747^b^
ESRS, mean scores ± SD		3.49 ± 5.15	3.09 ± 4.37	0.731^b^

^#^No *P* was significant.

^a^Fisher Exact Test.

^b^Two-tailed independent-samples *t*-test, equal variances assumed.

^c^Pearson chi-square. Abbreviations: PANSS, positive and negative syndrome scale; HDRS, Hamilton depression rating scale; and ESRS, extrapyramidal symptom rating scale.

### Schizophrenia-Related Symptoms

**Figure 2.**
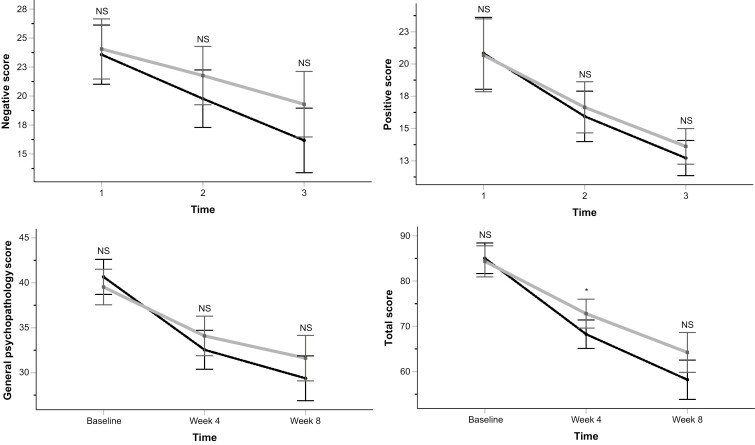
Repeated-measures analyses for comparison of the effects of risperidone + pentoxifylline (black dotted lines and circles) and risperidone + placebo (gray and solid lines and squares) on the Positive and Negative Syndrome Scale mean scores during the trial. Asterisk (*) shows a *P* <0.05 obtained from the independent-samples *t*-test comparing the scores at each time point between the groups, and NS shows nonsignificance.

Total and individual subscale scores of the PANSS at baseline did not differ between the groups (**Table 1**). **Table 2** provides a detailed breakdown of the changes in each subscale and total scores from baseline to each subsequent follow-up session. Additionally, **Figure 2** illustrates the statistical significance of comparing the values at each follow-up session between the groups.

**Table 2.** The changes in PANSS, HDRS, and ESRS scores from baseline to follow-up sessions.

Variable score changes		Time	Risperidone + pentoxifylline (n = 35), mean ± SD	Risperidone + placebo (n = 34), mean ± SD	MD (95% CI)	t	*P*	Cohen *d*
PANSS	Negative subscale	Until week 4	-3.80 ± 3.13	–2.29 ± 2.74	-1.51 (-2.92 to -0.09)	-2.124	0.037^a^^*^	0.512
		Until week 8	-7.40 ± 4.05	-4.76 ± 4.42	-2.63 (-6.67 to -0.60)	-2.583	0.012^a^^*^	0.622
	Positive subscale	Until week 4	-4.89 ± 4.73	-4.03 ± 4.50	-0.86 (-3.07 to 1.36)	-0.771	0.444^a^	0.186
		Until week 8	-8.11 ± 7.45	-7.06 ± 7.01	-1.05 (-4.53 to 2.42)	-0.605	0.547^a^	0.146
	General psychopathology subscale	Until week 4	-8.11 ± 7.38	-5.44 ± 5.89	-2.67 (-5.89 to 0.54)	-1.660	0.102^a^	0.400
		Until week 8	-11.29 ± 8.45	-7.91 ± 8.01	-3.37 (-7.33 to 0.58)	-1.701	0.094^a^	0.410
	Total	Until week 4	-16.77 ± 13.40	-11.59 ± 9.92	-5.21 (-10.87 to 0.45)	-1.840	0.070^b^	0.441
		Until week 8	-26.83 ± 17.16	-20.12 ± 17.04	-6.71 (-14.93 to 1.51)	-1.630	0.108^a^	0.392
HDRS		Until week 8	-0.43 ± 1.36	-0.53 ± 1.60	0.10 (-0.61 to 0.81)	0.283	0.778^a^	0.068
ESRS		Until week 8	-1.09 ± 2.76	-0.29 ± 2.19	-0.79 (-1.99 to 0.41)	-1.316	0.193^a^	0.317

^a^Two-tailed independent-samples *t*-test, equal variances assumed.

^*^Statistically significant.

^b^Two-tailed independent-samples *t*-test, equal variances not assumed. Abbreviations: ESRS, extrapyramidal symptom rating scale; MD, mean difference; PANSS, positive and negative syndrome scale; HDRS, Hamilton depression rating scale.

#### Negative Subscale Score—

There was a significant time effect on PANSS negative subscale scores (F = 115.429, df = 1.428, *P* <.001, ηP2 = .633 Greenhouse-Geisser corrected), showing that both groups experienced improvements in negative symptoms over the course of the trial. Additionally, there was a significant time–treatment interaction effect (F = 5.454, df = 1.428, *P* = .012, ηP2 = .075 Greenhouse-Geisser corrected), illustrating that the improvement rate varied between the groups (**Figure 2**), with the risperidone + pentoxifylline group showing significantly greater reductions until weeks 4 and 8 compared with the other group (**Table 2**).

**Table 3** presents the number of patients who achieved a response in steps of 25% PANSS negative subscale score reductions in each group at each follow-up session. By considering the 25% reduction as a priori, there was no significant difference in response rates between the groups until week 4 (37.1% vs 20.6%, respectively, *P* = .185). However, the risperidone + pentoxifylline group responded better by week 8 (82.9% vs 55.9%, respectively, *P* = .019). Additionally, the study found that the time required for patients to respond to treatment was slightly shorter in the risperidone + pentoxifylline group compared with the risperidone + placebo group, with means [95% CIs] of 6.207 [5.470-6.944] weeks compared with 6.526 [5.635-7.418] weeks, respectively (*P* = .587).

**Table 3.** The frequency of treatment responders in steps of 25% PANSS negative subscale score reductions between the groups until follow-up sessions.

Time	Group	˂25% reduction, n (%)	25-49.9% reduction, n (%)	50-74.9% reduction, n (%)	≥75% reduction, n (%)
Week 4	Risperidone + pentoxifylline (n = 35)	22 (62.9%)	9 (25.7%)	4 (11.4%)	0
	Risperidone + placebo (n = 34)	27 (79.4%)	4 (11.8%)	3 (8.8%)	0
Week 8	Risperidone + pentoxifylline (n = 35)	6 (17.1%)	10 (28.6%)	14 (40.0%)	5 (14.3%)
	Risperidone + placebo (n = 34)	15 (44.1%)	11 (32.4%)	7 (20.6%)	1 (2.9%)

Abbreviation: PANSS, positive and negative syndrome scale.

#### Other Scores—

Significant time effects on the PANSS positive and general psychopathology subscale scores (F = 65.451, df = 1.326, *P* <.001, ηP2 = .494 Greenhouse-Geisser corrected and F = 82.441, df = 1.184, *P* <.001, ηP2 = .552 Greenhouse-Geisser corrected, respectively), as well as total scores (F = 118.737, df = 1.151, *P* <.001, ηP2 = .639 Greenhouse-Geisser corrected) indicated improvements over time in these domains in both groups. Nonsignificant time–treatment interaction effects revealed that the groups did not differ regarding these domains over time (F = 0.354, df = 1.326, *P* = .614, ηP2 = .005 Greenhouse-Geisser corrected, F = 2.684, df = 1.184, *P* = .099, ηP2 = .039 Greenhouse-Geisser corrected, and F = 2.636, df = 1.154, *P* = .104, ηP2 = .038 Greenhouse-Geisser corrected, respectively) (**Figure 2**). Affirmatively, the changes in these domains did not differ between the groups until weeks 4 and 8 (**Table 2**).

None of the patients were in remission at baseline. By week 8, 13 (37.1%) participants in the risperidone + pentoxifylline group and 5 (14.7%) participants in the risperidone + placebo group achieved remission (*P* = .054).

### Depression Score

The HDRS scores at baseline did not differ between the groups (**Table 1**). Changes in HDRS scores from baseline to the end of the study were also comparable between the groups (**Table 2**).

### Safety and Tolerability

During the study, no severe complications that would have required participants to be excluded occurred. The baseline scores on the ESRS were similar between the groups (**Table 1**). The change in ESRS scores over the course of the study was also comparable between the groups (**Table 2**). Furthermore, no participants failed to adhere to their medication. Additionally, the frequency of adverse events did not differ between the groups (**Table 4**).

**Table 4.** The comparisons of side effect frequencies between the intervention arms.

Side effect	Risperidone + pentoxifylline (n = 37)	Risperidone + placebo (n = 37)	*P* ^a^ ^#^
Dizziness, n (%)	6 (16.2%)	4 (10.8%)	0.736
Eructation, n (%)	3 (8.1%)	1 (2.7%)	0.615
Bloating, n (%)	5 (13.5%)	4 (10.8%)	1.000
Abdominal pain, n (%)	4 (10.8%)	4 (10.8%)	1.000
Increased appetite, n (%)	4 (10.8%)	3 (8.1%)	1.000
Headache, n (%)	2 (5.4%)	1 (2.7%)	1.000
Diarrhea, n (%)	2 (5.4%)	2 (5.4%)	1.000
Nausea, n (%)	3 (8.1%)	4 (10.8%)	1.000
Vomiting, n (%)	2 (5.4%)	2 (5.4%)	1.000

^a^Fisher’s exact test.

^#^No *P* was significant.

## DISCUSSION

In this randomized, double-blind, placebo-controlled trial, the addition of pentoxifylline to the current risperidone treatment of 69 chronic schizophrenia patients resulted in the alleviation of negative symptoms during 8 weeks in a safe and tolerable manner. It also resulted in a better response up to the endpoint. These results were supported by the comparable baseline demographics and clinical features of the study arms.

Few studies have been conducted on the effects of pentoxifylline in the treatment of schizophrenia. A clinical trial was conducted in 2023 in which patients were treated with either 400 mg pentoxifylline or placebo twice a day for 8 weeks (Sinichi et al., 2023). There was an improvement in the PANSS positive subscale, but no significant changes were noted in negative symptoms. However, an increase in the number of Wisconsin Card Sorting Test categories was reported in the pentoxifylline group, potentially serving as an improved negative symptom indicator (Singh et al., 2017). The effects of pentoxifylline on negative symptoms of schizophrenia were also considered in a study, but that publication was retracted, and, consequently, the results were not compared. Other xanthine derivatives have also been studied in schizophrenia. The administration of 900 mg propentofylline added to risperidone in an 8-week, randomized, double-blind, placebo-controlled trial significantly affected positive and general psychopathology symptoms, unlike negative symptoms. Compared with the present study, the participants were chronic schizophrenia patients in their active phase who were not clinically stable (Salimi et al., 2008). Istradefylline is another xanthin derivative that enhanced the forced swim test, learned helplessness test, and motivation to work for reward test in different animal studies (Yacoubi et al., 2001; O’Neill and Brown, 2006; Yamada et al., 2014). These tests are associated with negative symptoms of schizophrenia, and the results are parallel to this study (Chatterjee et al., 2012; Martin et al., 2020; Monfort-Escrig and Pena-Garijo, 2021).

Management of negative symptoms in schizophrenia is of great importance due to its serious morbidities (Correll and Schooler, 2020). In the current study, pentoxifylline outperformed placebo in reducing negative scores. All participants engaged in this trial were clinically stable. Also, disorder duration, as well as HDRS, PANSS positive subscale, and ESRS scores, were recorded at baseline and endpoint to rule out the secondary sources of negative symptoms, including chronicity, depression, positive symptoms, and drug-induced akinesia (Peralta et al., 2000). The results showed no significant changes in the mentioned scales in the participants with chronic schizophrenia. By eliminating these factors, the distinct effect of pentoxifylline on primary negative symptoms could be studied.

Conversely, no significant differences in positive and general psychopathology symptoms were revealed between the groups. These findings may support the idea of risperidone sufficiency for targeting positive and general psychopathology symptoms in schizophrenia patients and the nonnecessity of combination therapy in this regard (Martinez-Gras et al., 2009; Shamabadi et al., 2023). Regarding positive symptoms, this can also be explained by the medication mechanism of action versus the neuropathophysiology of the disorder. Hyperactivity of dopamine transmission in mesolimbic areas and hypoactivity of dopamine transmission in the prefrontal cortex leads to positive and negative symptoms of schizophrenia, respectively (Brisch et al., 2014). As pentoxifylline increases dopaminergic neurochemical levels in the brain, pentoxifylline treatment may improve negative symptoms, unlike positive symptoms (Yu Wang et al., 2020). However, this cannot be generalized to general psychopathology symptoms due to the multiplicity of symptoms and complexity of the neuropathophysiology (McCutcheon et al., 2020).

The findings demonstrated that the addition of pentoxifylline specifically improved negative symptoms. Negative symptoms of schizophrenia are a consequence of reduced D1-receptor stimulation (Brisch et al., 2014). Pentoxifylline is a nonselective PDE inhibitor (Ward and Clissold, 1987). Former studies pointed out the potential role of PDEs in dopaminergic signaling pathways. PDE inhibition causes an increase in cAMP cellular levels and protein kinase A activation, eventually leading to stimulation of dopamine synthesis, inhibition of D2-receptors, and activation of D1-receptors (Nishi and Snyder, 2010). Moreover, PDE inhibitors exhibit antidepressant effects, which may help improve the negative symptoms associated with schizophrenia (Kaushik, 2011). Other PDE inhibitors have been studied in the treatment of negative symptoms of schizophrenia. A clinical trial reported the beneficial effects of an 8-week administration of cilostazol, a PDE3 inhibitor, as an adjunct to risperidone on negative symptoms (Rezaei et al., 2017). Also, 8 weeks of PDE5 inhibition with sildenafil administered adjunct to risperidone in another clinical trial had beneficial effects on negative symptoms (Akhondzadeh et al., 2011).

Inflammatory processes are also associated with negative symptoms. Evidence-based studies are representative of altered levels of inflammatory cytokines, including C-reactive protein, IL-1, IL-6, and TNF-α in schizophrenia patients, which affect the basal ganglia and induce negative symptoms (Stojanovic et al., 2014; Goldsmith et al., 2018; Liemburg et al., 2018). In addition, the addition of celecoxib to antipsychotic medication positively affected the negative symptoms (Müller et al., 2002), and adding pioglitazone to risperidone significantly decreased the PANSS negative subscale scores due to its anti-inflammatory and anti-oxidative effects (Iranpour et al., 2016). In these regards, pentoxifylline lessens the production of several cytokines, including those just mentioned (Fernandes et al., 2008). TNF-α, IL-1, and IL-6 secretions decreased considerably in human peripheral blood mononuclear cells after depletion of pentoxifylline (Neuner et al., 1994). A further study confirmed the efficiency of pentoxifylline in respiratory distress syndrome, which is a cytokine-driven disorder (Ardizzoia et al., 1993). Thus, the anti-inflammatory effect of pentoxifylline is another probable explanation for the results.

Assessing the regional cerebral blood flow (CBF) during the resting state via brain single photon emission computed tomography revealed inversed correlations between the number of negative subscale scores with frontal and prefrontal flows (Carol Sheei-Meei Wang et al., 2003), indicating the impact of reduced blood flow on negative symptoms. Bowton et al. proved increases in global and regional CBF after administration of 800 mg and 400 mg pentoxifylline in cerebrovascular patients, respectively (Bowton et al., 1989). Another trial reported a significant rise in CBF after an injection of 200 mg pentoxifylline in individuals with cerebrovascular disease (Koppenhagen et al., 1977), supporting the idea that pentoxifylline affects negative symptoms through its effect on blood perfusion.

The results indicated medium-sized effects of pentoxifylline on the primary outcome. Although adjusting the effect size range for subjective psychiatry scales would be more appropriate (Moncrieff and Kirsch, 2015), this effect in this short period could be of great interest considering the challenges posed by treating the negative symptoms (Correll and Schooler, 2020) and considering the participants were clinically stable.

Pentoxifylline as an adjunct to risperidone was well tolerated and safe in this study. In a previous study, no major side effects and extrapyramidal adverse effects were also reported after administrating this combination (Akhondzadeh et al., 2010).

This research presented notable advantages. A well-designed approach was applied to evaluate the primary negative symptoms by excluding the potential confounding factors. Also, the baseline characteristics of patients were accurately adjusted, and the same antipsychotic medication was administrated to all. Nevertheless, the current study encountered some limitations, including small sample size and short trial duration, as well as lacking post-intervention follow-up. Also, precise follow-up of patients was not applicable as they were not hospitalized during the investigation. Additionally, the evaluation of pentoxifylline treatment alone was not implemented due to ethical concerns. Finally, inflammatory biomarker levels were not measured in this study. Future research could incorporate objective measurements to better understand the connection between possible anti-inflammatory properties of pentoxifylline and the alleviation of negative symptoms.

In conclusion, pentoxifylline was beneficial, safe, and tolerated in the improvement of primary negative symptoms of chronic schizophrenia outpatients. The significant effects on a high-burden disorder within a restricted timespan are noteworthy. Additional research is required to validate the findings and rectify the limitations.

## Supplementary Material

pyae051_suppl_Supplementary_Appendix

## Data Availability

The data that support the findings of this study are available from the corresponding author upon reasonable request.

## Declarations

**Funding**

This study was supported by a grant from the Tehran University of Medical Sciences (TUMS) to Professor Shahin Akhondzadeh (grant no. 57532). TUMS had no role in the design, conduct, data collection, analysis, data interpretation, manuscript preparation, review, final approval, and the decision to submit the paper for publication.

## Clinical trial registration

The study protocol was registered and published in the Iranian registry of clinical trials (http://www.irct.ir; identifier IRCT20090117001556N143).
